# Trace amine-associated receptor 1 (TAAR1) agonists for psychosis: protocol for a living systematic review and meta-analysis of human and non-human studies.

**DOI:** 10.12688/wellcomeopenres.19866.1

**Published:** 2023-08-25

**Authors:** Spyridon Siafis, Robert McCutcheon, Virginia Chiocchia, Edoardo G. Ostinelli, Simonne Wright, Claire Stansfield, Damian Omari Juma, Ioannis Mantas, Oliver D. Howes, Grazia Rutigliano, Fiona Ramage, Francesca Tinsdeall, Claire Friedrich, Lea Milligan, Carmen Moreno, Julian H. Elliott, James Thomas, Malcolm R. Macleod, Emily S. Sena, Soraya Seedat, Georgia Salanti, Jennifer Potts, Andrea Cipriani, Stefan Leucht, the GALENOS team

**Affiliations:** 1 Department of Psychiatry and Psychotherapy, School of Medicine, Technical University of Munich, Munich, Germany; 2 Department of Psychiatry, University of Oxford, Oxford, England, UK; 3 Oxford Health NHS Foundation Trust, Oxford, England, UK; 4 Department of Psychosis Studies, Institute of Psychiatry, Psychology and Neuroscience, King's College London, London, England, UK; 5 Institute of Social and Preventive Medicine, University of Bern, Bern, Canton of Bern, Switzerland; 6 Oxford Precision Psychiatry Lab, University of Oxford, Oxford, England, UK; 7 Department of Psychiatry, Stellenbosch University, Stellenbosch, Western Cape, South Africa; 8 EPPI Centre, Social Research Institute, University College London, London, England, UK; 9 My Mind Our Humanity, Mombasa, Kenya; 10 Department of Clinical Neuroscience, Karolinska Institutet, Stockholm, Sweden; 11 Institute of Clinical Sciences, Faculty of Medicine, Imperial College London, London, England, UK; 12 Centre for Clinical Brain Sciences, The University of Edinburgh, Edinburgh, Scotland, UK; 13 MQ Mental Health Research, London, UK; 14 Department of Child and Adolescent Psychiatry, Institute of Psychiatry and Mental Health, Hospital General Universitario Gregorio Marañón, IiSGM, CIBERSAM, ISCIII, School of Medicine, Universidad Complutense de Madrid, Madrid, Community of Madrid, Spain; 15 Cochrane Australia, School of Public Health and Preventive Medicine, Monash University, Clayton, Victoria, Australia; 16 Future Evidence Foundation, Melbourne, Australia

**Keywords:** GALENOS; antipsychotic; neurotransmitters; pathophysiology; glutamate; schizophrenia; serotonin

## Abstract

Background: There is an urgent need to develop more effective and safer antipsychotics beyond dopamine 2 receptor antagonists. An emerging and promising approach is TAAR1 agonism. Therefore, we will conduct a living systematic review and meta-analysis to synthesize and triangulate the evidence from preclinical animal experiments and clinical studies on the efficacy, safety, and underlying mechanism of action of TAAR1 agonism for psychosis.

Methods: Independent searches will be conducted in multiple electronic databases to identify clinical and animal experimental studies comparing TAAR1 agonists with licensed antipsychotics or other control conditions in individuals with psychosis or animal models for psychosis, respectively. The primary outcomes will be overall psychotic symptoms and their behavioural proxies in animals. Secondary outcomes will include side effects and neurobiological measures. Two independent reviewers will conduct study selection, data extraction using predefined forms, and risk of bias assessment using suitable tools based on the study design. Ontologies will be developed to facilitate study identification and data extraction. Data from clinical and animal studies will be synthesized separately using random-effects meta-analysis if appropriate, or synthesis without meta-analysis. Study characteristics will be investigated as potential sources of heterogeneity. Confidence in the evidence for each outcome and source of evidence will be evaluated, considering the summary of the association, potential concerns regarding internal and external validity, and reporting biases. When multiple sources of evidence are available for an outcome, an overall conclusion will be drawn in a triangulation meeting involving a multidisciplinary team of experts. We plan trimonthly updates of the review, and any modifications in the protocol will be documented. The review will be co-produced by multiple stakeholders aiming to produce impactful and relevant results and bridge the gap between preclinical and clinical research on psychosis.

Protocol registration: PROSPERO-ID: CRD42023451628.

## Background and research questions

### Background

Psychotic disorders affect about 1% of the population and rank among the top 20 causes of disability worldwide ^1^. Antipsychotic drugs are the cornerstone of treatment and improve both acute psychotic symptoms, mainly in terms of positive symptoms (e.g., hallucinations and delusions), and also prevent relapses ^2, 3^. However, these medications are associated with multiple side-effects (e.g., weight gain and movement disorders) ^2^, high rates of non-response ^4^, and limited efficacy to negative symptoms (e.g., social-withdrawal and avolition) and cognitive impairment ^5^. Moreover, all currently licensed antipsychotics exert their clinical effects via antagonism of the dopamine D2 receptor (D2R) ^6^. Given the shortcomings of these treatments described above, there is an urgent need to develop treatments with novel mechanisms of action beyond the D2R antagonism.

A new approach is the agonism of trace amine-associated receptor 1 (TAAR1) ^7^. Trace amines, a group of monoaminergic neuromodulators serving as endogenous agonists for TAAR, share structural and metabolic similarities with classical monoamine neurotransmitters but are labelled as “trace” amines due to their significantly lower concentrations ^8^. TAAR is a family of G-protein coupled receptors (GPCR) discovered in 2001 in the search for novel receptors related to serotonin (5-HT) and dopamine receptors ^9, 10^. This family comprises 6 receptors in humans and 9 in rodents, which can be activated by endogenous trace amines, but also other related molecules and amphetamine-like psychostimulants ^11, 12^. Among them, TAAR1 has garnered significant attention as a promising and emerging target for mental health conditions, especially schizophrenia and other related psychotic disorders, since recent scientific investigations have put forth compelling evidence pointing to its pivotal role in the regulation of dopaminergic, glutamatergic and serotonergic neurotransmission ^11^. TAAR1 agonists are proposed to potentially possess efficacy across a wider spectrum of symptom domains than the current antipsychotics acting as D2R antagonists, including negative symptoms and cognitive impairment, while exhibiting a reduced propensity for side-effects ^11^. This was recently supported by a 4-week phase-II trial, where ulotaront (SEP-363856), an agonist of TAAR1 and the serotonin 1A receptor (5-HT1AR) with a negligible binding affinity to dopaminergic receptor, was found to be more efficacious than placebo in reducing overall symptoms of psychosis in individuals with acute schizophrenia while avoiding common side effects such as weight gain and movement disorders. It was, however, associated with a higher risk of gastrointestinal symptoms ^7^. According to another 6-week phase-II trial, ulotaront might be efficacious for the treatment of Parkinson’s disease psychosis without worsening the motor symptoms ^13^. However, two phase III trials investigating ulotaront for schizophrenia were recently announced to be negative, as they did not find differences from placebo, potentially due to high placebo responses ^14^.

There are currently multiple synthetic TAAR1 agonists in development in preclinical and/or clinical stages (e.g., ulotaront, RO5166017, RO5073012, RO5256390). There are also several important unanswered questions, such as the precise mechanism of action (e.g., including the role of serotonin and the interplay between presynaptic and postsynaptic mechanisms), the effects of TAAR1 agonists on negative symptoms and cognitive impairment, and the evaluation of long-term efficacy and side-effects ^11^. Therefore, we plan a living systematic review and meta-analysis, which goes beyond the scope of previous reviews that were primarily narrative, qualitative, static, or focused on a limited range of molecules (e.g., ulotaront) ^8, 11, 15^. Such an analysis would provide a multifaceted synthesis of the available evidence, incorporating the latest studies in this rapidly evolving field.

### Review objectives

- To synthesize and triangulate the evidence from preclinical animal experiments and clinical studies that investigate the efficacy, safety and the underlying mechanism of action of TAAR1 agonism for psychosis.

### Research questions

**For animal and preclinical studies:**

- What are the effects of TAAR1 agonists on *behavioural measures relevant to psychosis* in preclinical animal experiments of psychosis?
- What are the reported *side-effects* of TAAR1 agonists in preclinical animal experiments of psychosis?
- What are the effects of TAAR1 agonists on neurobiological measures relevant to psychosis such as *dopaminergic, glutamatergic and serotonergic signalling* in preclinical animal experiments of psychosis? Which are the *underlying molecular mechanisms* of these effects?
- If a *causal pathway* (or pathways) can be hypothesized based on the findings of the aforementioned research questions in earlier iterations of this living systematic review, is there any direct evidence available to support this hypothesis?

**For human studies:**

- What are the effects of TAAR1 agonists on the *symptoms of psychosis* in individuals with psychosis?
- What are the *tolerability and side-effects* of TAAR1 agonists in individuals with psychosis?
- What are the effects of TAAR1 agonists on neurobiological measures relevant to psychosis such as *dopaminergic, glutamatergic and serotonergic signalling* in individuals with psychosis? Which are the *underlying molecular mechanisms*?
- If a *causal pathway* (or pathways) can be hypothesized based on the findings of the aforementioned research questions in earlier iterations of this living systematic review, is there any direct evidence available to support this hypothesis?

## Methods of living systematic reviews

The project will be conducted within the GALENOS research program ^16^. The protocol is reported according to the GALENOS protocol template for living systematic reviews ^17, 18^ and the PRISMA statement for protocols (PRISMA-P) ^19^. The PRISMA-P checklist is provided as extended data ^20^. The protocol was registered with PROSPERO (ID: CRD42023451628) on 04.08.2023.

This is a ‘living systematic review’ in several respects, not just in the addition of new data as these become available. Thus, we plan an initial iteration of the review, in which we will apply narrower eligibility criteria, and future updates, in which we will apply broader eligibility criteria and more complex meta-analytic models (see extended data ^20^).

Given the “living” nature of this systematic review as well as the rapidly emerging evidence on TAAR1, changes in the protocol are expected, which will be clearly documented in future updated versions of the protocol (see “Updating the systematic review and stop the living mode of the review”).

### Study inclusion and exclusion criteria

The study inclusion and exclusion criteria are presented in Table 1 for animal studies and Table 2 for human studies.

**Table 1.** Inclusion and exclusion criteria for preclinical animal studies.

*Domains*	*Inclusion and exclusion criteria*
Study design	We will include controlled preclinical animal experiments investigating pharmacological TAAR1 agonism irrespective of the unit of allocation (e.g., individual animals or cage), parallel or crossover design, study duration and other methodological factors related to study quality and risk of bias (e.g., randomization, blinding of outcome assessment). There will be no other restriction in terms of the publication status (e.g., published in peer-reviewed journals, conference abstracts or as pre-prints, unpublished and obtained by personal communication or registries), language, year and country of origin. We will exclude *in vitro* and *in silico* studies, and uncontrolled experiments, for instance experiments where the animal serves only as its own control.
Animal population and model induction	We will include laboratory animals that have undergone any induction method pertaining to psychosis (“animal models of psychosis”) regardless of the age, sex, species, strain, and genetic composition (e.g., wildtype or genetic manipulation). There will be no restriction in terms of the induction method, as none of them can be considered gold standard ^29^. The different animal models of psychosis have unique strengths and weaknesses, and can be generally grouped into the following categories ^5, 29– 34^: 1) Pharmacological induction (e.g., administration of psychostimulants, NMDA antagonists or other psychotomimetic drugs). In particular, the “classic” pharmacological animal models of psychosis, i.e., induction with psychostimulants (e.g., amphetamine, apomorphine, and cocaine) and NMDA antagonists (e.g., ketamine, phencyclidine, and MK-801) have been widely employed in conjunction with their respective behavioural assays (see "Outcomes" in Table 1) in drug discovery for psychosis with a strong predictive validity in identifying the efficacy of antipsychotic medications ^5, 35, 36^. 2) Neurodevelopmental induction (e.g., gestational administration of MAM, post-weaning isolation, maternal immune activation). 3) Lesion induction (e.g., neonatal ventral hippocampal lesion in rats). 4) Genetic induction (e.g., DISC1 knockout, DAT knockout and D2R overexpression). 5) Any combination of the above induction methods. We will exclude animals that have not undergone an animal model of psychosis (e.g., “healthy” laboratory animals) and those animals that have undergone methods of induction for other specific conditions (e.g., valproic acid-induced model of autism) ^37^. However, if any of these models has been used by any author with a claim that it models aspects of psychosis, that model will be eligible for inclusion in *future updates* (see extended data ^20^).
Experimental interventions	We will include pharmacological agents that act as TAAR1 agonists, i.e., any ligands with evidence of inducing an active conformation of the TAAR1, irrespective of their pharmacological potency (e.g., half maximal effective concentration EC50) and efficacy (e.g., full or partial agonism), selectivity (e.g., affinities to other receptors like 5-HT1AR or D2R), dosing, timing, frequency, pharmacokinetic properties, route of administration (but which should be appropriate for achieving effects in the central nervous system) and co-administration with other agents (e.g., conventional antipsychotics). There is a growing number of synthetic TAAR1 agonists being developed (e.g., ulotaront, RO5166017, RO5073012, RO5256390, RO5203648, RO5263397, RO6889450) ^38^. We will identify agents acting as TAAR1 agonists by searching the literature ^8, 11^ and databases such as PDSP ^39^ and IUPHAR/BPS ^38, 40^. We will exclude from experimental interventions: 1) Amphetamine-like compounds (e.g., dexamphetamine, amphetamine, methamphetamine) and other psychotomimetic agents (e.g., LSD, psilocin), when they are identified as TAAR1 agonists ^8, 38, 40^, as these compounds have other primary mechanisms of actions (e.g., dopamine transporter inhibition or 5-HT2AR agonism) and are employed to induce animal models of psychosis (see “Animal population and model induction”) ^29, 31^. 2) Endogenous amines and related molecules acting as TAAR1 agonists (e.g., phenylethylamine, octopamine, tyramine, dopamine, 3- iodothyronamine), as these molecules participate in complex metabolic pathways exerting multiple actions. They will be considered in *future* *updates* (see extended data ^20^) 3) Pharmacological agents acting as TAAR1 antagonists (e.g., EPPTB), which will be considered as experimental intervention in *future updates* (see extended data ^20^), and those with a completely different mechanism of action (e.g., selective 5-HT2AR antagonists). 4) Genetic interventions targeting *TAAR1*, which will be considered in *future updates* (see extended data ^20^) *,* and those that do not target *TAAR1* (e.g., monoamine oxidase knockout - although experiments where these are used to induce a model of psychosis may qualify, see “Animal population and model induction”). 5) Other categories of interventions (e.g., brain stimulation, dietary intervention)
Control interventions	We will include multiple control conditions ^41^: 1) Vehicle, sham and untreated animal cohorts (the latter would be eligible when the more appropriate vehicle treatments are not available). There is a hierarchy in the above-mentioned control conditions: vehicle treatment involves the administration of a substance used to dilute the experimental intervention (e.g., injection of saline or mineral oil), sham treatment is designed to mimic the process of administering the experimental intervention (e.g., injection without the use of saline or the experimental intervention), and untreated animals where no intervention is being administered (apart from the method of induction as described in “Animal population and method of induction”) ^41^. 2) Animal cohorts receiving currently licensed antipsychotics will be considered as a positive control group, i.e., any drug with an Anatomical Therapeutic Chemical (ATC) code of N05A except for lithium. 3) TAAR1 antagonism via pharmacological or genetic manipulation. We will exclude uncontrolled experiments (see “Study design” in Table 1)
Outcomes	The co-primary outcomes will be the two most commonly used (identified from pilot searches) behavioural assays for psychotic (positive) symptoms, i.e., model-induced 1) locomotor hyperactivity and 2) impairment of prepulse inhibition (PPI) of the acoustic startle reflex. These behavioural assays have shown strong predictive validity in detecting the antipsychotic effects of drugs in the commonly employed pharmacological models of schizophrenia ^5, 30, 31, 33, 34^. However, their face validity for positive symptoms is generally limited. For instance, locomotor activity is an unspecific behaviour that more closely linked to psychomotor agitation, and PPI is more closely linked to sensorimotor gating than to positive symptoms, e.g., hallucinations and delusions. Thus, they can only be considered proxy markers for detecting positive symptoms ^35^. The secondary outcomes will be: 1) Other behavioural assays relevant to psychosis such as those related to positive symptoms (e.g., hallucination-like precepts) ^42^, negative symptoms (e.g., social interaction tests for social withdrawal, operant-based motivational tasks for avolition) ^35, 43^, cognitive impairment (e.g., tests recommended by CNTRICS like 5-choice serial reaction time task for attention, maze tests for learning and memory) ^34, 44, 45^, anxiety (e.g., elevation maze test, anxiety-like behaviors in open field test) ^31^ and depression (e.g., forced swim test for behavioral despair, sucrose preference test for anhedonia) ^35, 43^. 2) Adverse events (safety outcomes) including death, autonomic, metabolic, endocrine, neuromuscular, sensorimotor, and behavioural disturbance, which can be measured using batteries (e.g., functional observation battery and Irwin tests) ^46^ or other laboratory measurements (e.g., weight change, glucose and prolactin levels, temperature). 3) Neurobiological measures relevant to psychosis (mechanistic outcomes) such as measures of dopaminergic, glutamatergic and serotonergic signalling. There will be no restriction in terms of the timing outcome measurement and the specific testing paradigm of an assay (see “Data extraction”).

**Table 2.** Inclusion and exclusion criteria for human studies.

*Domains*	*Inclusion and exclusion criteria*
Study design	We will include clinical experimental studies investigating pharmacological TAAR1 agonism irrespective of the study duration, use of control (uncontrolled studies will also be eligible), phase (e.g., phase I-IV), parallel or crossover design, unit of allocation (e.g., individual patients or cluster), and factors related to study quality and risk of bias (e.g., randomization, blinding). There will be no other restriction in terms of the publication status (e.g., published in peer-reviewed journals, conference abstracts or as pre-prints, unpublished and obtained by personal communication or registries), language, year and country of origin. We will exclude non-experimental (i.e., observational) studies, *in vitro* and *in silico* studies.
Population	We will include individuals with a psychotic disorder for the investigation of efficacy outcomes, and individuals with or without a psychotic disorder (including healthy volunteers) for the investigation of safety and mechanistic outcomes (see below and “Outcomes”). For the investigation of efficacy outcomes, we will include individuals with psychosis irrespective of the underlying cause (e.g., schizophrenia spectrum disorders, psychosis related to dementia, Parkinson’s disease or substance use disorders). There will be no restriction in terms of age, sex, ethnicity, setting, diagnostic criteria used, baseline severity, existence of comorbidities, previous treatments, and duration of illness. Specific subpopulations will be eligible (e.g., acutely ill, stable, treatment-resistant and first-episode patients, and patients with predominant negative symptoms). When individuals with other mental health conditions (e.g., anxiety disorder) were included in a study, the latter will be eligible for the efficacy outcomes when the proportion of these individuals is less than 20% of the total sample. For the investigation of safety and mechanistic outcomes, we will include individuals with various mental health conditions (with or without psychosis) and healthy volunteers. Such broader inclusion criteria are generally recommended when investigating the potential adverse events of an intervention ^47, 48^. Furthermore, early phase and translational trials commonly include transdiagnostic samples and/or healthy volunteers ^49, 50^. Therefore, we will employ broader inclusion criteria for safety and mechanistic outcomes to enable a more comprehensive synthesis of the evidence pertaining to these outcomes. Given the broad inclusion criteria, we will consider subgroup and/or separate analyses of the specific diagnoses and subpopulations (see “Exploration of heterogeneity”), if applicable. We will exclude individuals who have solely physical health conditions without any accompanying mental health conditions, as mentioned above.
Experimental interventions	We will include pharmacological agents that act as TAAR1 agonists without any restriction as for “Experimental interventions” for animal studies in Table 1. We will exclude from experimental interventions amphetamine-like compounds and other psychotomimetic agents that may act as TAAR1 agonists, other pharmaceutical agents with a completely different mechanism of action (e.g., selective 5-HT2AR antagonists) and other categories of interventions (e.g., brain stimulation, dietary intervention) (see “Experimental interventions” for animal studies in Table 1).
Control interventions	We will include: 1) Placebo or no treatment. 2) Currently licensed antipsychotics will serve as a positive control group, i.e., any drug with ATC code of N05A except for lithium. 3) Single-arm studies, in which individuals receiving TAAR1 agonists serve as their own control (see “Study design” in eTable-2).
Outcomes	The primary outcome will be the severity of the overall psychotic symptoms as measured preferably by the Positive and Negative Syndrome Scale (PANSS) ^51^, and if not available, by the Brief Psychiatric Rating Scale (BPRS) ^52^ or any other validated scale ^53^. The secondary outcomes will be: 1) Severity of specific symptom domains (e.g., positive and negative symptoms, cognitive impairment, depressive symptoms) as measured by validated rating scales or tasks. 2) Clinically-important response to treatment (in case of acutely-ill patients) or relapse (in case of stable patients). 3) Dropouts due to any reason (as a measure of overall acceptability) and due to any adverse events (as a measure of overall tolerability). 4) Quality of life and global functioning as measured by validated rating scales. 5) Adverse events (safety outcomes), including any adverse event, death, serious adverse events and specific adverse events (e.g., autonomic, neuromuscular, metabolic, endocrine, behavioural). We will consider both the number of individuals with an adverse event (homogenized using the MedDRA® terminology) ^54^ and laboratory measures (e.g., weight change, QTc interval, prolactin levels). 6) Neurobiological measures relevant to psychosis (mechanistic outcomes) such as measures of dopaminergic, glutamatergic and serotonergic signalling. There will be no restriction in terms of the timing of the outcome measurement (see “Data extraction”).

It should be noted that the outcome section of the tables will not be considered in the eligibility criteria of the studies, i.e., studies will be included regardless of the outcome data reported.

### Study identification

The search strategies will be defined in collaboration with the search team. The ontology team will be informed of the search strategy and will help identify additional search terms where possible and relevant. The resulting search strategy will also inform the scope of the ontology. An ontology protocol will be available and will be included as supplementary content to the review (see extended data ^18^).

We will conduct independent searches for animal and human studies in multiple electronic databases to identify relevant records (titles/abstracts). For animal studies, we will search PubMed, Scopus, Web of Science and PsychINFO using search strategies compiled by keywords for TAAR1 and psychosis and appropriate filters for animal studies ^21^. For human studies, we will search PubMed/MEDLINE, Embase, International Pharmaceutical Abstracts, Web of Science, Biosis, PsychINFO, CENTRAL and Open Alex using search strategies compiled by keywords for TAAR1 and applying appropriate filters for human studies (psychosis terms will not be used, since individuals with other mental health conditions and healthy volunteers may also be eligible, Table 2). We will conduct searches from inception onwards, without applying any additional restrictions. The draft search strategies in PubMed/MEDLINE are provided in extended data ^20^, while similar search strategies will be developed for the other databases.

We will also search in registries of preclinical animal studies (e.g., animalstudyregistry.org, preclinicaltrials.eu) and clinical studies (e.g., clinicaltrials.gov and WHO-ICTRP) and inspect the references lists of eligible studies and previous reviews ^8, 11, 15^.

We will also contact pharmaceutical companies known to be investigating TAAR1 agonists (e.g., Roche and Sunovion) for additional animal and/or human studies.

The final search strategies in electronic databases will be reported according to the PRISMA statement for reporting literature searches (PRISMA-S) ^22^. The search strategies will be reviewed and revised, if it is deemed appropriate, before an update of the review (see “Updating the systematic review and stop the living mode of the review”). Moreover, we plan more comprehensive searches for unpublished studies and the integration of machine-assisted tools (e.g., psychosis-SOLES) ^23– 25^ in future updates of the review (see extended data ^20^).

### Study selection

The methodology of study selection and data extraction (see below) will be generally similar for animal and human studies.

The study selection will be performed using the tool of the Systematic Review Facility ( SyRF) ^26^ for animal studies and EPPI-Reviewer for human studies ^27^. The reviewers will be trained in a pilot phase by using a random sample of 30 title/abstracts and 5 full texts for both animal and human studies.

After electronic deduplication of the search results using the Automated Systematic Search Deduplicator (ASySD) ^28^ for the animal studies and EPPI-Reviewer for the human studies ^27^, the study selection will be conducted in two levels, i.e., title/abstract and full text.

Title/abstracts will be screened by at least two independent reviewers, and conflicts will be resolved by a third review that is blind to the decisions of the previous reviewers. Title/abstracts will be classified as “relevant” or “not relevant”, and “unclear” when it is not possible to judge the relevance of the record based on its title/abstract. Title/abstract screening will be offered until there is at least one reviewer and the agreement between the two reviewers is at least 0.65.

We will retrieve the full texts of “relevant” and “unclear” records from the first phase, which will be screened again by at least two independent reviewers for eligibility against the study inclusion and exclusion criteria. Again, conflicts between the two reviewers will be resolved by discussion with a third reviewer. If the full text is still unclear or if there is no available full publication of the record, we will contact the study authors to provide additional information. This step of evaluating the eligibility of the full-texts will be conducted independently and prior to the data extraction for human studies, but it will be carried out concurrently with the data extraction step for the animal studies.

The selection process for both animal and human studies will be recorded using the flow diagram structure of the extension of the PRISMA 2020 for living systematic reviews ^55– 57^. We will also present a table of excluded studies, which will refer to studies meeting the inclusion criteria but failed in one or more exclusion criteria. Moreover, we plan to utilize automated machine-assisted tools that would allow automated screening of the records with adequate performance in future updates of the review (see extended data ^20^).

### Data extraction

***Data extraction process***

Data extraction will be performed using standardized forms developed in the SyRF ^26^ for animal studies and EPPI-Reviewer for human studies ^27^. The data extraction forms will be sent to the ontology team so that relevant ontology categorisations can be identified to support data extraction. Moreover, the reviewers will be trained in the standardized forms and a pilot exercise will be performed in a random sample of 5 animal and 5 human studies.

At least two independent reviewers will perform the data extraction, and any disagreement will be resolved by discussion with a third reviewer. However, there will be one exception when it comes to extracting quantitative data from figures. Since it is unlikely for the extracted data to match precisely between the two reviewers, discrepancies exceeding 10% will be addressed through reconciliation. Otherwise, the mean value determined by the reviewers will be used for subsequent analysis.

We will consider multiple data sources for the data extraction according to the following hierarchy: i) text and tables, ii) figures (e.g., using the tool WebPlotDigitizer) ^58^, and in case of missing information iii) contacting authors, and iv) using imputation methods (see “Data items” below).

***Data items***

We will extract data related to study identification (e.g., first author, publication year, country of origin) and characteristics such as experimental design (e.g. unit and method of allocation), population (e.g., age, sex, species and method of induction for animal studies, diagnosis and patient subgroup for human studies), intervention (e.g., dose, route and timing of administration, duration of treatment) and control conditions (e.g., vehicle, sham, placebo and no-treatment, name and dose of antipsychotics), outcome measures (see also below for continuous and dichotomous outcomes) and risk of bias assessments (see “Risk of bias assessment”). In the initial iteration of the review, we will limit the extraction of data concerning study characteristics to the minimum necessary for data synthesis (see “Data synthesis”). However, as we progress to future updates of the review, we will expand the data extraction to provide a more comprehensive characterization of the included studies.

For continuous outcomes, we will extract the mean, standard deviation (SD), the number of persons/animals (Ns) and unit of measurement that these pertain. Missing SDs will be calculated from reported standard errors (SE), and if the latter is not available, they will be estimated according to the following hierarchy: from test statistics, e.g., p-values, t-tests, F-tests; confidence intervals and median/ranges ^59^; contacting authors or using a validated imputation method ^60^. If the measure of dispersion is unclear, i.e., whether it is SD or SE, we will contact the authors for clarification, and if we do not receive a response, we will make the conservative assumption that it is SE. Moreover, Ns are often not adequately reported in preclinical animal studies ^61^, and in that case, they will be estimated whenever possible, e.g., using the low boundary of a range. We will aim to extract baseline, endpoint and change scores from baseline at eligible timepoints (as described below), and preference will be given to change scores in the data synthesis. In addition, we will prefer to extract data from methods accounting for missing outcome data (e.g., mixed-models of repeated measurement (MMRM) and multiple imputation over last-observation carried forward (LOCF)) over observed cases. However, observed case data will also be eligible and missing outcome data will be considered in the risk of bias assessments (see “Risk of bias assessment”).

For dichotomous outcomes, we will extract the number of persons/animals with an event and the corresponding sample size from which these events were observed. For efficacy outcomes, we will use as the denominator the total sample of the study, assuming that persons lost to follow-up did not respond to the treatment (conservative assumption). For safety and mechanistic outcomes, we will use as the denominator the corresponding sample.

If an outcome is reported with both continuous and dichotomous measures (e.g., symptom improvement measured by mean score on a rating scale or number of responders based on a threshold score), preference will be given to the former.

In preclinical animal experiments, it is common to employ and report multiple tests or variations for the same outcome measure (e.g., multiple PPI assays with different pulse intensities). In such instances, we will extract data from all reported variations, including any correlation/covariance, as these data will be jointly synthesized (see “Data synthesis approach”).

In case of crossover trials, we will opt for using data from the first phase in order to avoid carryover effect ^62^. However, when data from the first phase are not available, we will consider using the data from the entire trial duration (i.e., before and after the crossover) by taking into account the within-subject correlation, which will be imputed when not explicitly reported (e.g., from t-tests or the literature) ^59^.

The timing of the outcome measure is contingent upon the specific research question within a study and cannot always be predetermined (see also “Exploration of heterogeneity”). In cases where the intervention is administered multiple times over an extended period, we will extract data at the following timepoints: 1) less than 3 weeks (preferably at the longest possible), 2) 3–13 weeks (preferably at 6 weeks – primary timepoint) and 3) longer than 13 weeks (preferably at the longest possible). This classification is particularly applicable to clinical trials investigating antipsychotics for acute episodes of psychosis ^2, 63, 64^, while longer-term outcomes are commonly observed in relapse prevention studies (e.g., after one or more years of treatment) ^3^. In cases where the intervention compromises a single or few doses administered and/or the outcome is measured within a 24-hour period and a monophasic response is expected (i.e., a rise to peak followed by a return to baseline), we will extract all available timepoints and calculate the mean area under the curve and its variance. This approach can be applicable to many of the preclinical animal experiments and early-phase translational trials.

***Risk of bias assessment***

We will evaluate the risk of bias (RoB) for the primary outcomes of animal and human studies. We will assess the risk of bias for the effects of assigning to the intervention, and we will consider the factors listed in the “Exploration of heterogeneity” as confounding domains in non-randomized trials.

We will use appropriate RoB tools to evaluate the biases in preclinical and clinical experiments, i.e., the SYRCLE’s tool for preclinical animal studies ^65^, the RoB2 tool for randomized controlled trials (RCTs) ^66^, and the ROBINS-I tool for non-randomized clinical trials ^67^. The completeness of reporting in terms of study design, conduct, and analysis is a prerequisite for assessing biases. However, the reporting of animal research is frequently incomplete, resulting in many publications being categorized as having an 'unclear' risk of bias in multiple aspects. As a result, we will also assess the quality of reporting in animal studies using an adapted extended version of the ARRIVE10 tool (see extended data) ^20, 68^.

Although the mentioned RoB tools assess similar categories of risk of bias (e.g., confounding, selection and information biases), they differ in their features and categorization. To ensure consistency and enhance the interpretability of the assessments, we will aim to harmonize the assessment and domains of bias across these different tools. These tools utilize signalling questions to evaluate the bias in different domains, assigning three or four levels of increasing risk ^65– 67^. The first three levels (low, moderate, and high risk) are consistent across the tools, while ROBINS-I includes an additional level of "critical risk," indicating a level of bias that renders the study unsuitable for inclusion in evidence synthesis ^67^. Moreover, we will note the possible direction of bias for each domain within a study whenever possible.

The judgments for each domain of bias will be combined to form an overall study-specific judgment using the following criteria: 1) If at least one of the domains is judged to have a "high" or "critical" risk of bias, the overall judgment for the study will be "high" or “critical” risk, respectively. 2) If at most one of the domains is judged to have a "moderate" risk of bias, the overall judgment for the study will be "low" risk of bias. 3) In all other cases, the overall judgement will be “some concerns” about bias.

If an RCT is assessed with a high risk of bias arising from the randomization process according to RoB2 ^66^, it will be classified as "non-randomized", and in such cases, its risk of bias will be evaluated using the ROBINS-I tool. If a non-randomized clinical trial is judged with an overall critical risk of bias, it will be considered too problematic and will be excluded from the evidence synthesis ^67^.

We will report the risk of bias judgements for each study. We will evaluate the impact of risk of bias by conducting a sensitivity analysis by restricting to studies with an overall low risk of bias (see “Sensitivity analyses”) *.* We will also use the risk of bias assessments to evaluate the confidence in the evidence (see “Summary of the evidence”).

### Data analysis and synthesis

We will synthesize separately the data from animal and human studies, and their findings will be jointly interpreted using triangulation methods (see “Triangulation of the evidence from living systematic reviews”).

***Comparison of study findings and synthesis***

**Effect sizes**

The effect sizes for continuous outcomes will be the mean difference (MD) when outcomes are measured on the same scale/unit across all studies (e.g., kg for weight or other laboratory values), and the standardized mean difference (SMD, Hedge’s g) when outcomes are measured on different scales/units (e.g., behavioural measures). For preclinical animal studies, we will consider a sensitivity analysis using normalized mean differences (NMD) when outcomes are measured on different scales/units and the performance of untreated animals can be known or inferred in the majority of the studies. We plan this sensitivity analysis because variances can be small (or even zero) in preclinical animal experiments, especially when the group size is very small, and in that case SMDs cannot be calculated ^61^. In case of single-arm studies, we will calculate absolute or standardized mean changes from baseline for continuous outcomes ^69, 70^. We will apply minus transformations, whenever appropriate, to ensure that they correspond to the same direction (e.g., scores >0 indicating improvement). Along with the previous effect sizes, we will also calculate the variability ratio (VR) or the coefficient of variability ratio (CVR), in case of a mean-variance relationship in order to provide additional insights into the reproducibility and generalizability of the findings ^71^.

The effect size for dichotomous outcomes will be odds ratio (OR) due to their preferred mathematical properties in meta-analysis ^72^. Natural logarithms of ORs will be used in the meta-analysis and they will be back-transformed for presentation. If a meta-analysis is possible (see below “Data synthesis approach”), we will also convert the pooled ORs from the meta-analysis to relative and absolute risks in order to ease the interpretability of the findings ^73^. This conversion would require an assumption for the control event rate (CER), which will be the point estimate of a single-group random-effect meta-analysis of the vehicle/sham/placebo control groups. In case of single-arm studies, we will calculate the proportion of participants with an event (logit-transformed in the meta-analysis) ^74^.

When outcomes can be reported with both continuous and dichotomous measures (see “Data extraction”), we will also consider transforming odds ratios to continuous measures (e.g., SMDs) using the Hasselblad and Hedges method in order to allow a more comprehensive synthesis of the evidence ^75– 77^.

The effect sizes will be presented along with their 95% confidence intervals (95%CI).

Last, we will consider unit of analysis issues (e.g., allocation by clusters, repeated measures or shared control) and adjust the study estimates accordingly ^59^, such as with a multilevel meta-analytic model (see below) ^78^.

**Comparisons**

We will investigate the following comparisons: 1) TAAR1 agonists versus vehicle, sham, placebo or no-treatment (for both animal and human studies), 2) TAAR1 agonists versus currently licensed antipsychotics (for both animal and human studies), 3) TAAR1 agonists versus TAAR1 antagonism (for animal studies) and 4) pre-post changes in individuals receiving TAAR1 agonists (only for human studies).

In future updates, we will consider utilizing a network meta-analysis to offer a more elaborated synthesis of the evidence on the comparative effects of the experimental and control interventions (see extended data) ^20, 79^.

**Data synthesis approach**

We will opt to conduct meta-analysis whenever possible, but if the available data are deemed unsuitable, we will consider synthesis without meta-analysis (SWiM) ^80^. This will be examined by visually inspecting the forest plots considering the direction and magnitude of effects, the degree of overlap between 95%CIs across the individual studies.

When meta-analysis is deemed appropriate, we will employ a random-effects meta-analysis within a frequentist framework. For preclinical animal studies, we will use a multilevel multivariate meta-regression model with robust variance estimation (RVE) to allow a flexible handling of non-independent data and the decomposition of variance components (e.g., clustering of animal cohorts) ^78, 81^. We will include covariates in the random-effects structure for the study record (or laboratory in case that multiple experiments come from the same laboratory), species (or strain in case only rodents are available), method of induction, cohort of animals (in case multiple effect sizes are available for the same animal cohort, see “Data extraction”) and the specific measurement of the outcome (in case various measurements are available for the same outcome, see “Data extraction”). We will consider the available data and levels of covariates when building the model (rule of thumb of at least 5 levels for a random-effects covariate) ^78, 82^. Moreover, in case of non-independent sampling errors (e.g., multiple effect sizes for the same animal cohort), we will estimate the within-study variance-covariance matrix (VCV) using the reported correlations/covariances in a study (see “Data extraction”), and when not available, using an assumed correlation of ρ=0.5 (ρ=0.2 and ρ=0.8 in “Sensitivity analysis”) ^78^. Other potential sources of heterogeneity will be investigated with meta-regressions (see “Exploration of heterogeneity”).

We will use the restricted maximum likelihood (REML) to estimate the between-study variance (τ ^2^) and the between-study VCV in multivariate meta-analytic models (for animal studies) ^83^. We will apply Hartung-Knapp method to adjust the confidence intervals of the treatment effects if there are at least 5 studies ^84^. Heterogeneity will be quantified using the τ ^2^ and the 95% prediction intervals (95%PI) of the treatment effects.

**Software**

Data cleaning and analysis will be conducted in R statistical software using the packages tidyverse ^85^, meta ^86^, metafor ^70^ and clubSandwich ^87^.

***Exploration of heterogeneity***

If a meta-analysis is possible and there are sufficient data, we will examine potential study characteristics as source of heterogeneity for the primary outcomes in subgroup (meta-regression) analysis. We will opt for multivariable meta-regression models, but in case the amount of data is not sufficient, we will conduct exploratory univariable meta-regressions.

We will investigate the following characteristics for both animal and human studies (unless otherwise specified): 1) age, 2) sex, 3) species/strain (only for animal studies), 4) method of induction (only for animal studies), 5) baseline severity, 6) diagnosis and patient subgroups (only for human studies), 7) dose of the TAAR1 agonist, 8) potency (e.g., based on the half maximal effective concentration, EC50) and efficacy (e.g., full or partial) of the TAAR1 agonist, 9) selectivity of the intervention in terms of TAAR1 agonism (e.g., accompanied 5-HT1R agonism, co-treatment with antipsychotics), 10) duration of treatment (see “Data extraction”).

It should be noted that time-course and dose-effects can be important potential effect-modifiers and will also be considered in the assessment of the confidence in the evidence (see “Summary of the evidence”). However, it would be difficult to predefine the methodology of assessing time-course and dose-effects given the potential substantial differences across species and pharmacological agents (e.g., differences in potency and efficacy, as described above). Therefore, we plan to conduct subgroup analysis to examine these, but any specific decision will be indicated a posteriori. Moreover, we will opt to apply time-course and dose-response meta-analysis in future updates (extended data) ^20, 88– 90^.

***Sensitivity analyses***

If a meta-analysis is possible, we will examine the robustness of the findings for the primary outcomes by 1) restricting the analysis to studies with an overall low risk of bias, 2) excluding estimates with imputed values (e.g., SDs, Ns), 3) using NMD as the effect size (for animal studies only) and 4) sampling correlations of ρ=0.2 and ρ=0.8 to construct the within-study VCV matrices (for multivariate meta-analysis, see “Data synthesis”).

### Reporting bias

We will examine both within- and across-study reporting bias and assess the potential impact on the magnitude or direction of the findings. We will opt for using existing tools such as the preliminary tool for assessing risk of bias of missing evidence (ROB-ME) ^91^. However, as mentioned above, we will not actively search for unpublished studies in the first iteration of the review, except for unpublished trials in registries (see “Study identification”).

We will also examine small-study effects for the primary outcome when there are more than 10 available studies by visually inspecting contour-enhanced funnel plots ^92^ and conducting an regression-based tests ^93^ or it’s the extension of Egger’s regression test for multilevel meta-analysis ^78^. We will consider potential reasons of small-study effects such as heterogeneity and publication bias.

### Summary of the evidence

We will evaluate the confidence in the summary of the evidence using an adapted version of the GRADE framework for both animal and human studies ^94, 95^, irrespective of the use of a meta-analysis or SWiM for data synthesis. The evaluation will take into account the summary of the association (e.g., magnitude and direction of the effects, imprecision and heterogeneity), potential concerns in terms of internal and external validity of the including studies, potential biases in the review process (“meta-bias”) and reporting biases. The importance of these issues in determining the confidence in the evidence will be assessed by a single reviewer by assigning “no concerns”, “some concerns” or “major concerns”, and the judgements will be verified by a second reviewer.

We will present the assessments and judgements in summary of evidence (SoE) tables for each outcome, by presenting in the rows the different sources of evidence (e.g., animal and human studies) and in the columns the different domains relevant to the confidence of the evidence.

In the first iteration of the review, we will specifically evaluate the confidence in evidence for the *primary outcomes* that are relevant for the first review question, i.e., *the effects of TAAR1 agonists on psychotic symptoms and their behavioural proxies* (see “Research questions”). Table 3 presents the structure of the SoE tables and the domains that will be considered for this research question.

**Table 3.** Summary of Evidence (SoE) table for the effects of TAAR1 agonists on psychotic symptoms and their behavioural proxies (primary outcomes).

*Source of the* *evidence*	*Summary of the* *association (magnitude* *and direction of the* *effects, imprecision and* *heterogeneity)*	*Internal validity (within-* *study bias)*	*External validity* *(indirectness and/or* *translatability)*	*Reporting bias and other* *sources of meta-bias*
Clinical studies for the effects on psychotic symptoms (separately for the different comparisons)
	Number of studies and total sample size. Point estimate, 95%CI and 95%PI, or SWiM range. Distribution of the effect sizes across the individual studies.	Percentage of studies with low, moderate or high risk of bias (see “Risk of bias assessment”). We will consider the overall judgement, the judgements across domains and the potential direction of bias (e.g., towards the null or to any direction). Assessment of the robustness of the findings with a sensitivity analysis restricting to studies with an overall low risk of bias (see “Sensitivity analysis”).	Assessment of the degree to which the characteristics of the included studies reflect the clinical setting (see above) ^94– 97^. We will also consider the potential direction of the bias in case of indirectness. Meta-analysis of variation, as a low inter-individual variability could suggest findings that are more generalizable and reproducible (see “Effect sizes”) ^71^.	Assessment of the potential impact of reporting bias on the magnitude and direction of the findings using the ROB-ME tool ^91^. *In the first iteration*, the search will not be exhaustive, as we will not actively pursue unpublished studies, except for searching registries (see “Reporting bias”). No other sources of meta-bias are expected, as we will follow a rigorous review methodology aimed at minimizing biases in the review process ^98^.
Preclinical animal experiments for the effects on behavioral proxies of psychotic symptoms (separately for the two co-primary outcomes, and the different comparisons)				

## Triangulation of the evidence from living systematic reviews

Preclinical animal experiments and clinical studies consist of distinct sources of evidence with unique systematic biases that will be documented in SoE tables (see “Summary of the evidence”). Therefore, we will use triangulation methods to interpret their findings together and draw an overall conclusion.

The potential of triangulation will be assessed based on the amount of available evidence for at least one outcome and from at least two sources of evidence (see “Summary of the evidence”), and it will be assessed in every update of the review. In the first iteration of the review, we will consider the triangulation of the evidence for the primary outcomes that are relevant for the first review question, i.e., *the effects of TAAR1 agonists on psychotic symptoms in clinical studies and their behavioural proxies in preclinical animal experiments*.

If triangulation is appropriate, we will organize a "triangulation meeting" consisting of a multidisciplinary team (e.g., epidemiologists, systematic review methodologists, psychiatrists, neuropsychopharmacologists) in order to ensure the inclusion of essential expertise required for effective triangulation, i.e., methodological expertise in evidence synthesis of preclinical animal experiments and/or clinical studies, methodological expertise in preclinical animal experiments, clinical studies and/or translational research in psychosis, and content expertise in antipsychotics, psychosis and/or TAAR1.

The aims of the “triangulation meeting” will be to evaluate the confidence of the evidence for each source of the evidence (rows in SoE tables) by discussing and taking into consideration the direction, impact and sources of biases (columns in SoE tables) as well as any information about dose-effects relationships (e.g., based on the dose and pharmacological potency and efficacy of TAAR1 agonists, see “Exploration of heterogeneity”), and draw an overall conclusion from the SoE table about the effects of TAAR1 agonists on psychotic symptoms.

At the end of a triangulation meeting, the multidisciplinary team will assess whether the objectives and research questions of the review have been adequately addressed based on the conclusions from the SoE tables and the overall findings of the review. If yes, the team will decide whether to stop the living mode of the review. If not, the team will identify the potential need to update or revise the methods or the focus of the review (see “Updating the systematic review and stop the living mode of the review”).

## Updating the systematic review and stop the living mode of the review

**Figure 1.**
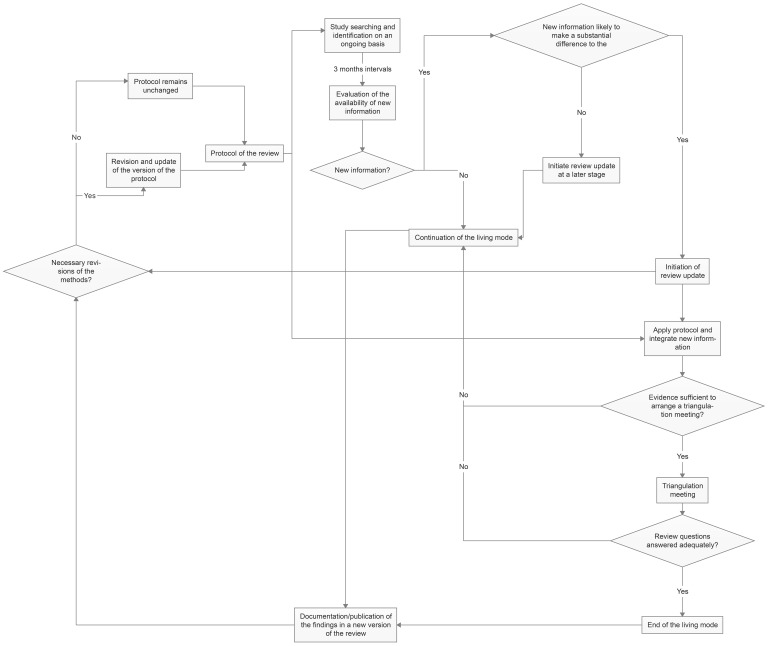
Flow diagram of the living systematic review and protocol.

The process of updating the systematic review is presented in Figure 1.

We plan to update the search of the living systematic review on an ongoing basis, potentially utilizing a combination of automated searches, machine learning, and crowdsourcing. Specific methods are yet to be determined.

Every *3 months*, we will assess the availability of new information identified through the ongoing study search and identification process. If this new information is likely to make a substantial difference to the findings of the the review (e.g., direction of effects, point estimates, precision of estimates, potential need for a triangulation meeting), we will initiate an update of the review. However, if there is no new information or the new information is not expected to substantially alter the review findings, we will not initiate an update and will prioritize other competing living systematic reviews of the GALENOS project ^16^.

In the event of initiating a review update, we will examine the necessity for a triangulation meeting (refer to "Triangulation of the evidence from living systematic reviews"). If a triangulation meeting takes place, we will assess whether the objectives and research questions have been adequately addressed to consider stopping the living mode of the review (refer to "Triangulation of the evidence from living systematic reviews").

If an update of the review is not initiated or a triangulation meeting does not occur, the living mode will continue by default, and the evaluation of new information will be conducted in intervals of 3 months, as mentioned above.

Furthermore, before and after each update, we will also consider whether the methods require updating and revision. This may include expanding the inclusion criteria, conducting a more comprehensive search, considering more complex meta-analytic methods, or broadening the list of primary outcomes. Any updates in the review protocol (e.g., revision of the methods) and the review itself (e.g., implementation of new data) will be clearly documented, and a detailed versioning system will be used.

The living systematic review will use a versioning system based on the one used by F1000 and that any deviations from the methods outlined in this protocol will be documented and justified.

## Co-production aspects

We have employed a multidisciplinary approach by considering the perspectives, experience and knowledge of multiple stakeholders such as preclinical and clinical researchers, clinicians, systematic review methodologists, statisticians, and experiential advisors. This approach would be crucial in producing highly relevant results for the community and bridging the preclinical-clinical disconnection in research on psychosis ^5^.

In formulating the focus of the review, we drew upon existing prioritization exercises that incorporated co-production in their process, i.e., the UK Mental Health Research Goals 2020–2023 ^99^, the WHO Grand Challenges in Mental Health ^100^, and the James Lind Alliance's Top 10 Priorities for depression ^101^ and schizophrenia ^102, 103^. Through these exercises, common themes emerged, such as the need for research to develop new and improved treatments, understand the root causes of mental health conditions, and gain a better understanding of the therapeutic mechanisms underlying current drug and psychological treatments. These themes provided the foundation for the initial research questions within GALENOS.

To ensure the comprehensive consideration of perspectives from all stakeholders involved, we will assemble a team of co-authors who represent the diverse backgrounds mentioned above. It is anticipated that each co-author will make a more substantial contribution to specific sections based on their individual experiences and expertise. The review team will receive guidance from the work package 1 (WP1) of GALENOS on effective models of involvement for Experiential Advisors ^16^. As a result, a multidisciplinary approach will be implemented throughout all stages of the review, from the identification of needs, the formulation of the research aims, the design of the review, and the interpretation and dissemination of the findings to the research and public community.

Considering the complexity and multidimensionality of the review topic, we will establish a schedule of regular team meetings and foster effective communication within the GALENOS project. The primary objective of these initiatives is to facilitate a shared understanding, promote the transferability of knowledge, encourage the exchange of ideas and perspectives, and identify the distinct needs of various stakeholders. By implementing these measures, we aim to create an environment where all stakeholders have equal standing and can actively contribute to the collaborative production of the review.

## Dissemination of information

We plan to publish the review on the GALENOS website and on Wellcome Open Research. A Plain English summary will accompany the review. We will use social media outlets (Twitter, Facebook) to publicise the results and will write blog posts that will be available on the GALENOS website. We will also include the results in the quarterly Research Roundup newsletter that MQ issues. We hope to present GALENOS at the World Congress of Biological Psychiatry as well as other conferences.

## Study status

The study status at the date of submission 04.08.2023 is reported below.

### Preliminary searches

Started, but not completed.

### Piloting the study selection process

Not started.

### Piloting the study selection process

Not started.

### Full searches

Not started.

### Full screening of search results against eligibility criteria

Not started.

### Data extraction

Not started.

### Risk of bias or quality assessment

Not started.

### Data synthesis

Not started.

## Data Availability

No data are associated with this article. Open Science Framework: Trace amine-associated receptor 1 (TAAR1) agonists for psychosis: protocol for a living systematic review and meta-analysis of human and non-human studies, https://doi.org/10.17605/OSF.IO/86Z2P ^20^. Open Science Framework: GALENOS, https://doi.org/10.17605/OSF.IO/WMGDQ ^18^. (also CC-BY 4.0). This project contains the following extended data: Adapted version of ARRIVE 10.pdf Brief ontology protocol.pdf Methods for future updates.pdf Search strategies.pdf Open Science Framework: PRISMA-P checklist for ‘Trace amine-associated receptor 1 (TAAR1) agonists for psychosis: protocol for a living systematic review and meta-analysis of human and non-human studies.’, https://doi.org/10.17605/OSF.IO/86Z2P ^20^. Data are available under the terms of the Creative Commons Attribution 4.0 International license (CC-BY 4.0).
