# Supplementary material for: Trace amine-associated receptor 1 (TAAR1) agonists for psychosis: protocol for a living systematic review and meta-analysis of human and non-human studies
Source: Wellcome Open Res. 2023 Aug 25;8:365. [Version 1] doi: 10.12688/wellcomeopenres.19866.1 (PMC11021884; doi:10.12688/wellcomeopenres.19866.1)
Supplement: Peer review history

# Reviewer report: Reviewer response for version 1

**DOI:** 10.21956/wellcomeopenres.21999.r74762

**Published:** 2024-04-16

**Authors:** Andreas S Lappas

**Affiliations:** 1 University of Thessaly, Larissa, Greece

Thank you for the opportunity to review this study protocol. It is very well written and very comprehensive. Just a few minor comments which may need further elaboration. 

1. Duration of treatment: for clinical populations - should there be a set minimum acceptable duration of treatment? Especially when comparing with antipsychotics, what would the minimum acceptable duration of antipsychotic treatment be?
2. For add on studies, would there be any requirements regarding duration of stable treatment that the agent is added on to? What if this is not mentioned specifically? How is this going to be handled? This may not only be important for psychotropics that the agent is added on for treatment of neuropsychiatric symptoms, but also say for treatment of motor symptoms of IPD, i.e. dopamine agonists. Also, how would the authors handle studies that may not specify the treatment that TAAR1 agonists are added on to? For example, they may report add-on to antipsychotics without specifying which.
3. Subgroup analysis: could the authors please elaborate more on the clinical categories they will investigate? They mention diagnoses and subgroups but they don't elaborate more. 
4. Treatment resistant schizophrenia and first episode psychosis - what would the criteria for defining these be? I presume these would be groups included in the subgroup analysis? It would be clinically very useful to include these. 
5. They mention they will consider NMA so transitivity may be a concern due all to the above. 
6. Sensitivity analysis: would the authors consider exclusion of studies sponsored by the pharmaceutical industry and studies from mainland China ^1-3^? 

Is the study design appropriate for the research question?

Yes

Is the rationale for, and objectives of, the study clearly described?

Yes

Are sufficient details of the methods provided to allow replication by others?

Yes

Are the datasets clearly presented in a useable and accessible format?

Not applicable

Reviewer Expertise:

Clinical psychiatry, meta-research in psychiatry

I confirm that I have read this submission and believe that I have an appropriate level of expertise to confirm that it is of an acceptable scientific standard.

## References

[1] : An assessment of the quality of randomised controlled trials conducted in China. *Trials* .2008;9: 10.1186/1745-6215-9-22 22 10.1186/1745-6215-9-22 18435861 PMC2373774

[2] : Quality assessment of reporting of randomization, allocation concealment, and blinding in traditional Chinese medicine RCTs: a review of 3159 RCTs identified from 260 systematic reviews. *Trials* .2011;12: 10.1186/1745-6215-12-122 122 10.1186/1745-6215-12-122 21569452 PMC3114769

[3] : 80% of China's clinical trial data are fraudulent, investigation finds. *BMJ* .2016;355: 10.1136/bmj.i5396 i5396 10.1136/bmj.i5396 27707716

# Reviewer report: Reviewer response for version 1

**DOI:** 10.21956/wellcomeopenres.21999.r68125

**Published:** 2023-11-06

**Authors:** Giuseppe De Simone

**Affiliations:** 1 Section of Psychiatry, Laboratory of Translational and Molecular Psychiatry and Unit of Treatment-Resistant Psychosis, Department of Neuroscience, Reproductive Sciences and Dentistry, University Medical School of Naples "Federico II", Naples, Italy

The present study aims to evaluate the effect of TAAR1 agonism from clinical and preclinical perspectives. Overall, the protocol is well-written and comprehensive. I have only three concerns:

1. The authors assert that there will be no limitations in terms of publication status. Studies that compared treatment estimates of meta-analyses with and without conference abstracts reported changes in precision, and, in some cases, differences in the estimate of the treatment effect ^1^. Thus, if authors were to include conference abstracts as well as other papers not published in peer-reviewed journals, it would be fair to conduct a sensitivity analysis.
2. High heterogeneity is present among preclinical models of psychosis. Attention should be paid not only to the type of model but also to the characteristics of each. In pharmacological induction models, different results could be obtained depending on the age at which the drug is administered and the paradigm used (acute, subchronic, or chronic administration). 
3. The authors will include compounds with slightly different pharmacodynamic properties such as ulotaront, RO5166017, RO5073012, and RO5256390. Do they provide for separate analyses?

Is the study design appropriate for the research question?

Yes

Is the rationale for, and objectives of, the study clearly described?

Yes

Are sufficient details of the methods provided to allow replication by others?

Yes

Are the datasets clearly presented in a useable and accessible format?

Not applicable

Reviewer Expertise:

My area of expertise is psychotic disorders, especially from a preclinical perspective.

I confirm that I have read this submission and believe that I have an appropriate level of expertise to confirm that it is of an acceptable scientific standard.

## References

[1] : How should systematic reviewers handle conference abstracts? A view from the trenches. *Syst Rev* .2019;8(1) : 10.1186/s13643-019-1188-0 264 10.1186/s13643-019-1188-0 31699124 PMC6836535
